# Supplementary material for: Prognostic Significance of TWIST1, CD24, CD44, and ALDH1 Transcript Quantification in EpCAM-Positive Circulating Tumor Cells from Early Stage Breast Cancer Patients
Source: Cells. 2019 Jun 29;8(7):652. doi: 10.3390/cells8070652 (PMC6679222; doi:10.3390/cells8070652)
Supplement: Supplementary file 1 [file cells-08-00652-s001.zip › cells-529153 supplementary/Supl Figure 2.pptx]

## Slide 1
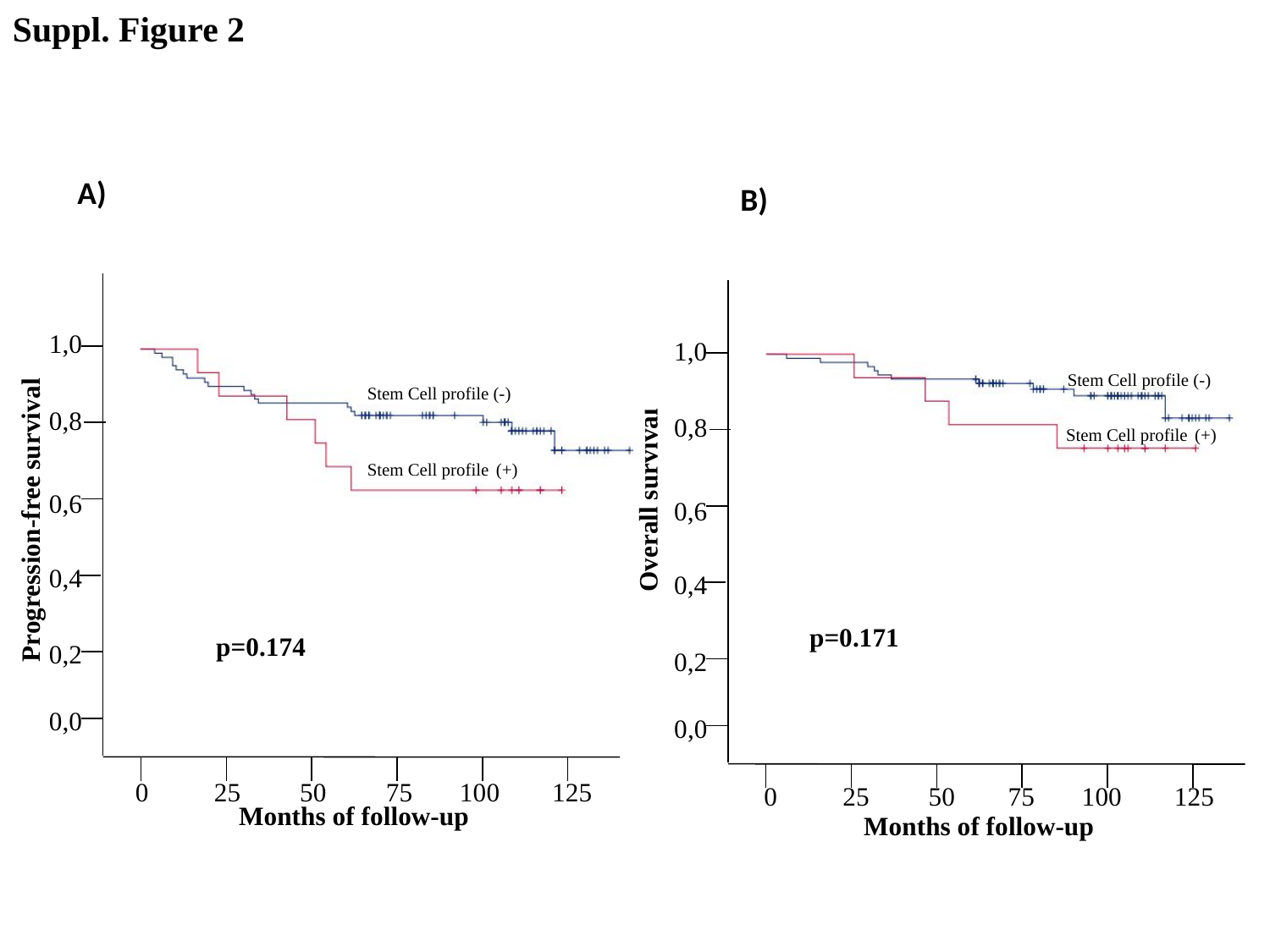

Suppl. Figure 2
A)
B)
Overall survival
1,0
0,8
0,6
0,4
0,2
0,0
Months of follow-up
Progression-free survival
1,0
0,8
0,6
0,4
0,2
0,0
Months of follow-up
Stem Cell profile (-)
Stem Cell profile (-)
Stem Cell profile (+)
Stem Cell profile (+)
p=0.171
p=0.174
0 25 50	 75	 100 125
 0 25 50	 75	 100 125

## Slide 2
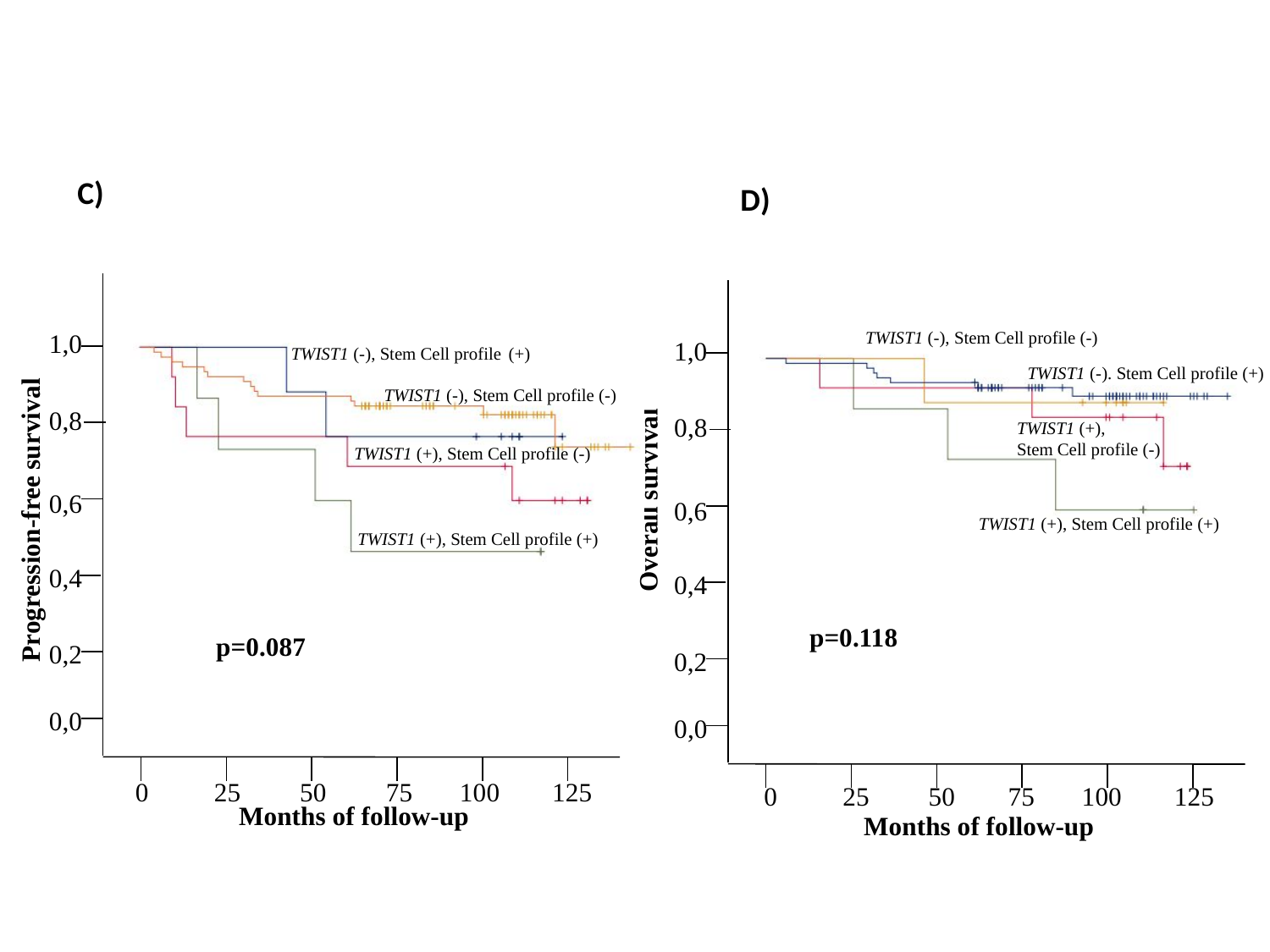

C)
D)
Overall survival
1,0
0,8
0,6
0,4
0,2
0,0
Months of follow-up
Progression-free survival
1,0
0,8
0,6
0,4
0,2
0,0
Months of follow-up
TWIST1 (-), Stem Cell profile (-)
TWIST1 (-), Stem Cell profile (+)
TWIST1 (-). Stem Cell profile (+)
TWIST1 (-), Stem Cell profile (-)
TWIST1 (+),
Stem Cell profile (-)
TWIST1 (+), Stem Cell profile (-)
TWIST1 (+), Stem Cell profile (+)
TWIST1 (+), Stem Cell profile (+)
p=0.118
p=0.087
0 25 50	 75	 100 125
 0 25 50	 75	 100 125
