# Supplementary material for: Prognostic Significance of TWIST1, CD24, CD44, and ALDH1 Transcript Quantification in EpCAM-Positive Circulating Tumor Cells from Early Stage Breast Cancer Patients
Source: Cells. 2019 Jun 29;8(7):652. doi: 10.3390/cells8070652 (PMC6679222; doi:10.3390/cells8070652)
Supplement: Supplementary file 1 [file cells-08-00652-s001.zip › cells-529153 supplementary/Supplemental Tables.pdf]

Supplemental Tables

Supl. Table 1. Clinical characteristics of the patients with early breast cancer (n=105).

**Qualitative Variable**		**Stem Cell Profile** $CD44^{high}/CD24^{-/low}$ $ALDH1^{high}/CD24^{-/low}$		**Mesenchymal Profile** *TWIST1*		**Stem Cell and mesenchymal Profile** $CD44^{high}/CD24^{-/low}$ $ALDH1^{high}/CD24^{-/low}$ *TWIST1*	
**Patients enrolled**	**All n (%)**	**Positive n (%)**	**Negative n (%)**	**Positive n (%)**	**Negative n (%)**	**Positive n (%)**	**Negative n (%)**
	100(100)	15(15.0)	85(85.0)	19(19.0)	81(81.0)	7(7.0)	93(93.0)
**Menopausal**							
Pre	91(91.0)	14(15.4)	77(84.6)	16(17.6)	75(82.4)	6(6.60)	85(93.4)
Post	9(9.0)	1(11.1)	8(88.9)	3(33.3)	6(66.7)	1(11.1)	8(88.9)
		N.S		0.366		0.494	
**Age, years**							
≥ 50	50(50.0)	7(14.0)	43(86.0)	9(18.0)	41(82.0)	4(8.0)	46(92.0)
<50	50(50.0)	8(16.0)	42(84.0)	10(20.0)	40(80.0)	3(6.0)	47(94.0)
		N.S		N.S		N.S	
**T Stage**							
T1	34(34.0)	5(14.7)	29(85.3)	3(8.8)	31(91.2)	2(5.9)	32(94.1)
T2/T3	66(66.0)	10(15.2)	56(84.8)	16(24.2)	50(75.6)	5(7.6)	61(92.4)
P		NS		0.063		0.753	
**Grading**							
1/2	34(34.3)	7(20.6)	27(79.4)	5(14.7)	29(85.3)	3(8.82)	31(91.2)
3	65(65.7)	8(12.3)	57(87.7)	13(20.0)	52(80.0)	4(6.20)	61(93.8)
Missing values	1	0.275		0.514		0.689	
**LN**							
N0	36(37.1)	5(13.9)	31(86.1)	3(8.33)	33(91.7)	2(5.6)	34(94.4)
N1	35(36.1)	4(11.4)	31(88.6)	7(20.0)	28(80.0)	1(2.90)	34(97.1)
N2/N3	26(26.8)	6(23.1)	20(76.9)	9(34.6)	17(65.4)	4(15.4)	22(84.6)
Missing values	3	0.437		**0.036**		0.155	
**ER**							
Positive	62(63.9)	10(16.1)	52(83.9)	12(19.3)	50(80.6)	4(6.4)	58(93.5)
Negative	35(36.1)	5(14.3)	30(85.7)	6(17.1)	29(82.8)	3(8.6)	32(91.4)
Missing values	3	0.640		0.640		0.7	
**PR**							
Positive	51(52.6)	9(17.6)	42(82.3)	10(19.6)	41(80.4)	4(7.80)	47(92.2)
Negative	46(47.4)	6(13.0)	40(87.0)	8(17.4)	38(82.6)	3(6.5)	43(93.5)
Missing values	3	0.585		0.8		N.S	
**HER2**							
Positive	38(39.6)	4(10.8)	34(89.2)	2(5.40)	36(94.6)	1(2.7)	37(97.3)
Negative	58(60.4)	10(15.9)	48(84.1)	16 (25.4)	42(74.6)	6(9.5)	52(90.5)
Missing values	4	0.362		**0.006**		0.238	

Supl. Table 2 : quadraplex RT-qPCR for *CD24*, *CD44*, *ALDH1*, *HPRT*: Evaluation of intra- (n=3) and inter-assay (n=5) precision.

CD24		***Intra-assay precision (n=3)***		
**SKBR-3 cells**	**Cq (SD)**	**CV%**	**Copies (SD)**	**CV%**
**1**	26.80 (0.12)	0.45	1.6 (±0.15) x 10^2	10
**10**	23.33 (0.25)	1.1	3.3 (±0.72) x 10^3	23
**100**	20.33 (0.19)	0.93	4.4 (±0.72) x 10^4	18
**1000**	16.97 (0.087)	0.51	801 (±0.62) x 10^5	7.7
		Inter-assay precision(n=5)		
**1000 SKBR-3**	16.99 (0.19)	1.1	8 (±0.13) x 10^5	16

CD44		***Intra-assay precision (n=3)***		
**SKBR-3 cells**	**Cq (SD)**	**CV%**	**Copies (SD)**	**CV%**
**1**	31.68 (0.026)	0.082	0.81 (±0.021)	2.6
**10**	28.72 (0.28)	0.97	1.5 (±0.38) x 10	26
**100**	25.50 (0.35)	1.4	3.5 (±1.1) x 10^2	30
**1000**	21.84 (0.15)	0.69	1.2 (±0.18) x 10^4	15
		Inter-assay precision(n=5)		
**1000 SKBR-3**	21.56 (0.20)	0.93	1.6 (±0.32) x 10^4	20

ALDH1		***Intra-assay precision (n=3)***		
**SKBR-3 cells**	**Cq (SD)**	**CV%**	**Copies (SD)**	**CV%**
**1**	31.64 (0.16)	0.51	0.82 (±0.12)	15
**10**	28.19 (0.067)	0.24	2.4 (±0.16) x 10	6,6
**100**	25.07 (0.23)	0.92	5.1 (±1.2) x 10^2	24
**1000**	21.72 (0.15)	0.69	1.3 (±0.20) x 10^4	14
		Inter-assay precision (n=5)		
**1000 SKBR-3**	21.39 (0.18)	0.84	1.9 (±0.33) x 10^4	18

HPRT		***Intra-assay precision (n=3)***		
**SKBR-3 cells**	**Cq (SD)**	**CV%**	**Copies (SD)**	**CV%**
**1**	30.64 (0.21)	0.68	8.4 (±0.16)	20
**10**	28.32 (0.25)	0.88	8.0 (±0.20) x 10	25
**100**	25.59 (0.23)	0.90	1.0 (±0.27) x 10^3	24
**1000**	21.97 (0.24)	1.1	3.7 (±0.18) x 10^4	23
		Inter-assay precision (n=5)		
**1000 SKBR-3**	21.71 (0.18)	0.83	4.7 (±0.81) x 10^4	17

Supl. Table 3. Correlation of *TWIST1*, *CD44*^{high}/*CD24*$^{-/low}$ and/or *ALDH1*^{high}/*CD24*$^{-/low}$ and the co-expression of *TWIST1* and *CD44*^{high}/*CD24*$^{-/low}$ and/or *ALDH1*^{high}/*CD24*$^{-/low}$ with the patients' clinical outcomes.

Qualitative Variable	Stem Cell Profile<br>*CD44*^{high}/*CD24*$^{-/low}$<br>*ALDH1*^{high}/*CD24*$^{-/low}$		Mesenchymal Profile<br>*TWIST1*		Stem Cell and mesenchymal Profile<br>*CD44*^{high}/*CD24*$^{-/low}$<br>*ALDH1*^{high}/*CD24*$^{-/low}$<br>*TWIST1*	
Recurrence	***Positive***	***Negative***	***Positive***	***Negative***	***Positive***	***Negative***
Yes	6(24.0%)	19(76.0%)	9(36.0%)	16(64.0%)	4(16.0%)	21(84.0%)
No	9(12.0%)	66(88.0%)	10(13.3%)	65(86.7%)	3(40.0%)	72(96.0%)
P[a]	0.194		**0.019**		0.063	
Death	***Positive***	***Negative***	***Positive***	***Negative***	***Positive***	***Negative***
Yes	4(28.6%)	10(71.4%)	6(42.9%)	8(57.1%)	3(21.4%)	11(78.6%)
No	11(12.8%)	75(87.2%)	13(15.1%)	73(84.9%)	4(4.7%)	82(95.3%)
P[a]	0.217		**0.024**		0.055	

[a] Fisher's Exact Test
