# Supplementary figures and images for: Prognostic Significance of TWIST1, CD24, CD44, and ALDH1 Transcript Quantification in EpCAM-Positive Circulating Tumor Cells from Early Stage Breast Cancer Patients
Source: Cells. 2019 Jun 29;8(7):652. doi: 10.3390/cells8070652 (PMC6679222; doi:10.3390/cells8070652)

## Slide 1
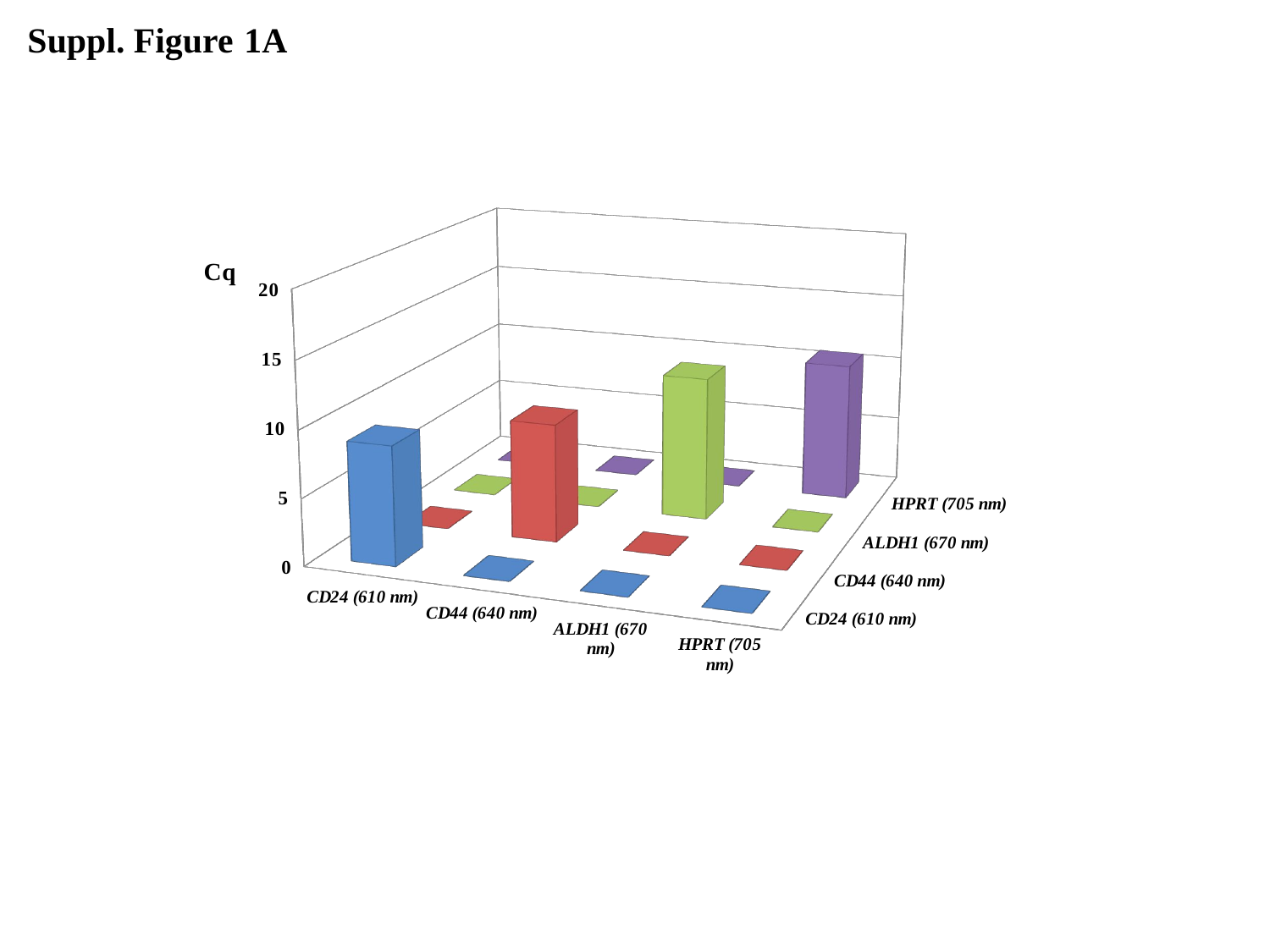

Suppl. Figure 1A
[unsupported chart]

## Slide 2
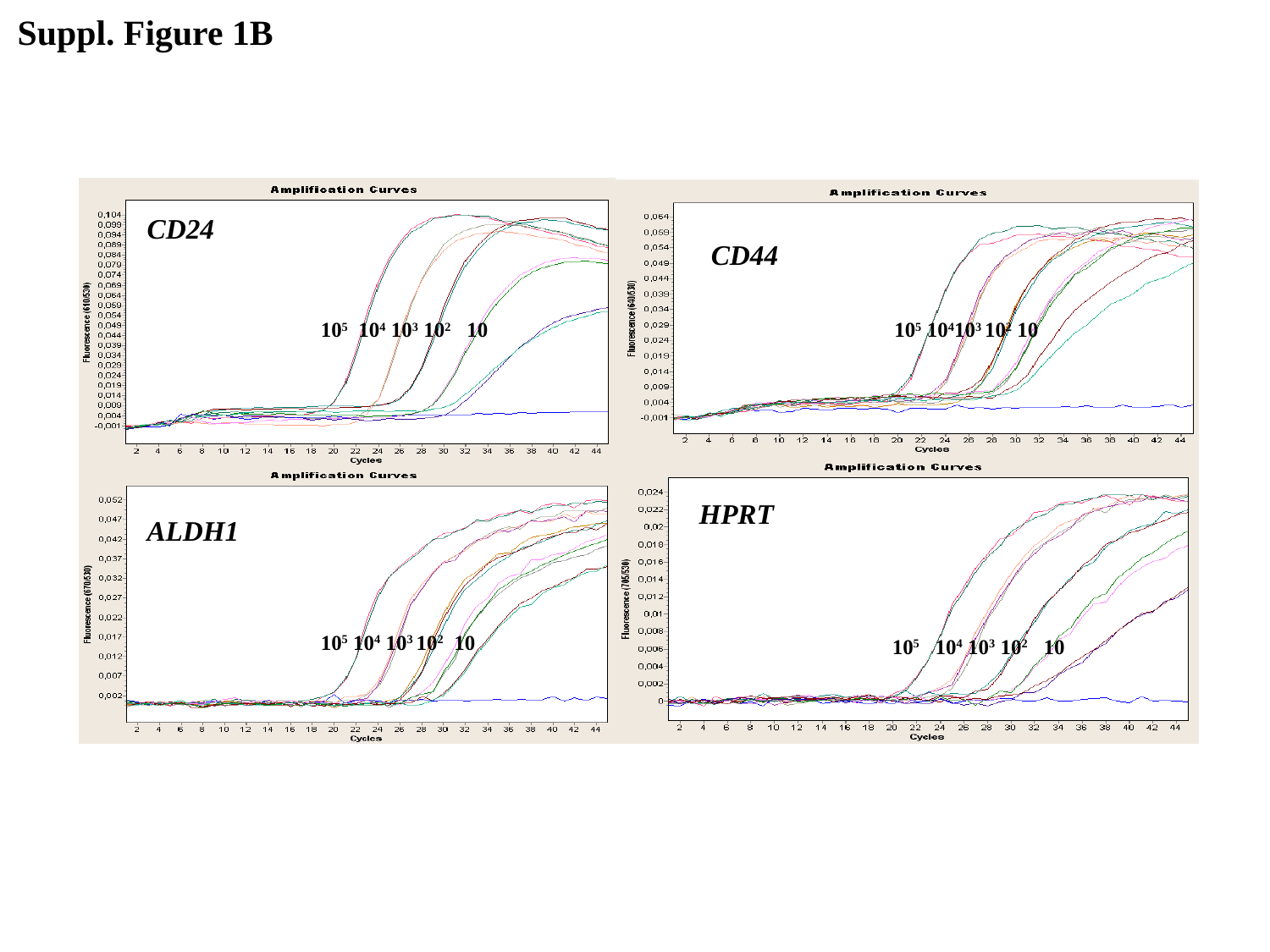

Suppl. Figure 1B
CD24
CD44
105 104 103 102 10
105 104103 102 10
HPRT
ALDH1
105 104 103 102 10
105 104 103 102 10

Supplement: Supplementary file 1 [file cells-08-00652-s001.zip › cells-529153 supplementary/Supl Figure 1.pptx]
